# Response to ‘Interspinous bursitis is common in polymyalgia rheumatica, but is not associated with spinal pain’

**DOI:** 10.1186/s13075-015-0620-7

**Published:** 2015-04-28

**Authors:** Peter A Simkin

**Affiliations:** University of Washington, Box 356428, Seattle, WA 98195 USA

In a recent contribution to this journal,Camellino and colleagues [1] reported that asymptomatic inflammation of the interspinous bursas is a common occurrence in patients with polymyalgia rheumatica. Thus, they confirm the findings of three other studies which found florid inflammation (which was felt to be symptomatic) at these sites between the spinous processes of adjacent vertebrae. Why would this be so when bursitis at more accessible locations is not recognized as a part of this syndrome? One possible explanation might be found in the proximity of each interspinous bursa to its adjacent ligamentum flavum [2]. If the fundamental process is an auto-immune response to elastic tissue, then this most elastic of all anatomical structures would be an appropriate target and a 'sympathetic effusion' might be expected in adjacent tissues such as the interspinous bursas.

A similar situation could explain the characteristic synovitis of large joints in polymyalgia. There, scanning techniques demonstrate an unequivocal inflammatory cause for the joint pain and stiffness, but the effusions are small, synovial fluid findings are unimpressive, and the synovitis is not destructive. This, too, could represent an immune response to aging elastic tissue of the joint capsule with the synovitis being secondary.

Possible elastic antigenicity is not a new idea in giant cell arteritis [3]. Localization of inflammatory cells around the internal elastic laminas of temporal artery biopsies was prominent from the first recognition of this entity and remains so today [4]. Apparent elastic fragments within giant cells, immunoglobulin deposits adjacent to the internal elastic lamina, proliferative responses of lymphocytes to arterial tissue *in vitro*, and a striking response of mononuclear cells to elastin peptides all lend support to this idea, although none of these findings have been replicated convincingly [5].

Polymyalgia rheumatica can be excruciating, giant cell arteritis can be lethal, and both conditions respond dramatically to appropriate therapy. Yet their diagnosis can be difficult, and their common link has not been found. Confirmation of elastic antigenicity would provide much-needed understanding of the basic pathophysiology and could thereby facilitate both diagnosis and management. Does not someone want to take another look at the possibility that these findings and concepts may be held together with an elastic band?
